# Physical and Mechanical Characterization of Titica Vine (*Heteropsis flexuosa*) Incorporated Epoxy Matrix Composites

**DOI:** 10.3390/polym13234079

**Published:** 2021-11-24

**Authors:** Juliana dos Santos Carneiro da Cunha, Lucio Fabio Cassiano Nascimento, Fernanda Santos da Luz, Sergio Neves Monteiro, Maurício Ferrapontoff Lemos, Cristina Gomes da Silva, Noan Tonini Simonassi

**Affiliations:** 1 Department of Materials Science, Military Institute lof Engineering—IME, Praça General Tibúrcio, 80, Urca, Rio de Janeiro 22290-270, Brazil; julianasccunha@gmail.com (J.d.S.C.d.C.); lucio@ime.eb.br (L.F.C.N.); fsl.santos@gmail.com (F.S.d.L.); 2 Brazilian Navy Research Institute—IpqM, Materials Technology, Rua Ipiru, 02, Cacuia, Rio de Janeiro 21931-095, Brazil; mauricio.lemos@marinha.mil.br; 3 Department of Materials Science, Federal University of Amazonas—UFAM, Avenida General Rodrigo Octávio Jordão Ramos, 1200-Coroado 1, Manaus 69067-005, Brazil; cristinagomes.ufam@gmail.com; 4 Advanced Materials Laboratory (LAMAV), Department of Materials Engineering, State University of the Northern Rio de Janeiro—UENF, Avenida Alberto Lamego, 2000, Campos dos Goytacazes 28013-602, Brazil; noantoninisimonassi@gmail.com

**Keywords:** titica vine fiber, epoxy composite, natural fiber, chemical treatment, mechanical properties

## Abstract

Titica vine (*Heteropsis flexuosa*) is a typical plant of the Amazon region commonly used for making baskets, bags, brooms and furniture, owing to its stiff fibers. In spite of its interesting properties, there is so far no reported information regarding the use of titica vine fibers (TVFs) in engineering composite materials. In this work, the TVF and its epoxy composites were for the first time physically, thermally and mechanically characterized. Additionally, the effect of two kinds of chemical treatments, one with sodium carbonate and one with calcium lignosulfonate, as well as different volume fractions, 10, 20, 30 and 40 vol%, of TVF-reinforced composites were assessed for corresponding basic properties. The thermogravimetric results of the composites reveal enhanced thermal stability for higher TVF content. In addition, the composite incorporated with 40 vol% of TVFs treated with sodium carbonate absorbed 19% more water than the composites with untreated fibers. By contrast, the calcium lignosulfonate treatment decreased water absorption by 8%. The Charpy and Izod impact tests showed that the composites, incorporated with the highest investigated volume fraction (40 vol%) of TVF, significantly increased the absorbed energy by 18% and 28%, respectively, compared to neat epoxy. ANOVA and Tukey statistical analyses displayed no direct influence of the chemical treatments on the energy absorption of the composites for either impact tests. SEM images revealed the main fracture mechanisms responsible for the performance of TVF composites.

## 1. Introduction

In recent years, factors such as increasing environmental awareness, new regulations and unsustainable petroleum consumption have driven the promotion of natural materials as substitutes for synthetic ones [1,2]. In this context, the incorporation of natural lignocellulosic fibers (NLFs) into polymeric matrices gained a recognized prominence in the area of composite materials, whether in research works [3,4,5] or industrial applications [6,7,8,9]. Much of this interest is related to the NLFs’ recyclability, biodegradability, low-density and cost-benefit characteristics [8,10,11,12]. In addition, their relevant properties are able to combine with those of polymer matrices and produce reinforced composites that exhibit relatively higher physical and mechanical strength.

In spite of many advantages, NLFs have some limitations. One of them is related to their thermal degradation. In fact, decomposition of the main components of natural fibers (cellulose, lignin and hemicellulose) starts at relatively low temperatures, around 200 °C, this being the main limiting factor for the processing and application of NLF-reinforced composites [13,14]. Another drawback is associated with NLFs’ hydrophilic nature, which contrasts with the hydrophobic character of most polymeric matrices. A strong fiber/matrix interfacial bond is responsible for better adhesion between phases and therefore plays a predominant role in the characterization of the mechanical properties of composites [15,16,17,18]. To overcome the problems of affinity in the interface zone, the surface of NLF can be modified through physical treatments, such as electric discharge and thermal exposure, as well as chemical treatments associated with alkaline, acetylation, peroxide and silane surface modifications [5,17,18,19,20].

Although alkaline treatment with NaOH is well consolidated and has shown good results [21,22,23,24], factors such as concentration and exposure time may have a negative influence if not properly dosed [25,26]. In addition, it is interesting to look for treatments involving reagents from renewable sources. Sodium carbonate (Na_2_CO_3_) is an easy-to-purchase, low-cost and less dangerous product as compared to NaOH, and still has lower degradation behavior towards the fibers [27]. Calcium lignosulfonate (CaLS), on the other hand, is a derivative of lignin obtained from the pulping process of sulphite [28,29]. The objective of the treatment with CaLS was to combine the polar and non-polar groups common not only to NLF and polymer resin but also to the lignosulfonate itself, allowing a greater compatibility between the matrix and the reinforcement, and consequently better adhesion at the interface.

**Figure 1:**
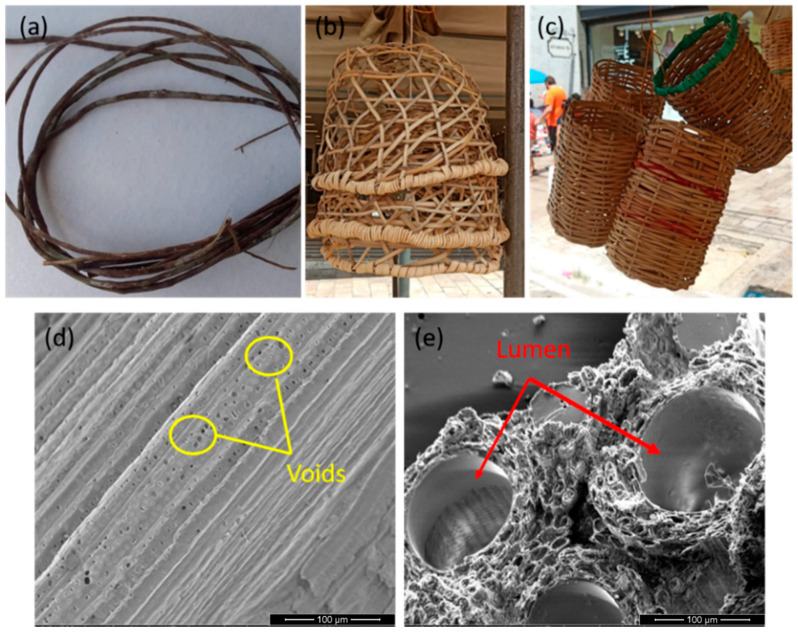
(**a**) Titica vine root taken from the Amazon Forest; (**b**,**c**) baskets made from titica vine fiber (TVF); (**d**) SEM image of longitudinal section of the TVF indicating voids and pores; (**e**) SEM image of cross-section of the TVF indicating regions of the lumen.

The titica vine fiber (TVF), extracted from the plant botanically called *Heteropsis flexuosa*, is a relatively unknown NLF. Figure 1a illustrates the collected titica vine root from the Amazon forest together with simple baskets made of TVFs in Figure 1b,c, as well as the scanning electron microscopy (SEM) details of a TVF surface in Figure 1d,e. Peoples of the South American rainforest use TVFs to fabricate handicrafts, baskets and furniture [30]. However, unlike the more common and recently exploited Amazon fibers such as curaua [31], buriti [32], mallow [33], guaruman [34] and others, there are no reports in the literature about TVF’s properties as a reinforcing agent in composite materials. In the present study, TVFs were modified using two reagents: Na_2_CO_3_ and CaLS, for better adherence to epoxy matrix composites.

Therefore, this work, for the first time, investigates the physical and mechanical behavior of epoxy composites incorporated with TVFs both untreated and treated by modification with Na_2_CO_3_ and CaLS. Water absorption, thermal analysis and Izod and Charpy impact tests were performed, as well as scanning electron microscopy on the fracture surface.

## 2. Materials and Methods

### 2.1. Materials

**Figure 2:**
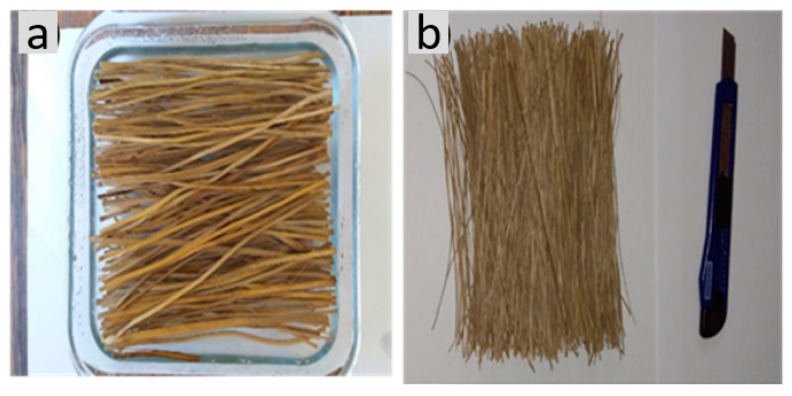
Extraction process of TVFs: (**a**) roots in water-immersed splints; (**b**) manual extraction of the fibers.

As illustrated in Figure 2, the titica vine roots were acquired in a local market in the city of Boa Vista, state of Roraima, north of Brazil. Splints were mechanically cut from each root and immersed in water for 24 h to facilitate the extraction of the fibers, as shown in Figure 2a. Titica vine fibers (TVFs) were manually extracted from each splint with a blade, as shown in Figure 2b.

The fibers were subjected to treatments with Na_2_CO_3_ (≥99% purity) supplied by Quimisul (Santa Catarina, Brazil) and CaLS (≥99% purity) from Auro’s Quimica (Sao Paulo, Brazil). The fibers were initially washed at 70 °C with water, followed by washing with 99.3% ethylic alcohol (Prolink quimica, Sao Paulo, Brazil) and acetone P.A (95:5 *v*/*v*) from BHerzog (Rio de Janeiro, Brazil), and then were heating for 1 h at 70 °C. The treatment with Na_2_CO_3_ was based on the work of Santos et al. [27]. A total of 16.7 wt% of TVFs were immersed in 10 wt% Na_2_CO_3_ solution (200 g TVFs per 1000 mL Na_2_CO_3_ solution), with a pH of about 12 per 24 h at room temperature (RT). Subsequently, the fiber samples were washed in running water for 20 min and dried in a stove at 60 °C for 24 h following similar procedure elsewhere [27]. Another portion of the same percentage of fibers, as in the previous treatment, was soaked in 5 wt% CaLS solution and subjected to a temperature of 70 °C and magnetic agitation. After this period, they were removed and dried again in the oven, as described elsewhere [35]. 

The possible effect of these treatments in terms of the percent of TVF that is functionalized by Na_2_CO_3_ and CaLS could in principle be investigated by techniques such as X-ray photoelectron spectroscopy (XPS) or fluorescence (XRF). Although not a main objective of the present work, preliminary XRF results conducted in a model Epsilon 3-XLE PANalytical spectrometer (PANalytical, Malvern, Worcestershire, UK) suggest that only ∼10% of the TVF might have been functionalized by the Na_2_CO_3_, while around 80% was functionalized by CaLS. More reliable results are expected to be obtained by XPS in our ongoing research work.

A commercial epoxy resin, diglycidyl ether of bisphenol A (DGEBA)-type, hardened with triethylenetetramine (TETA), associated with a stoichiometric ratio of 13 parts hardener to 100 parts of resin, was used as the polymeric matrix. Both resin and hardener were supplied by Epoxy Fiber, Rio de Janeiro, Brazil. Among the polymers, epoxy is one of the most commonly used matrices in composites incorporated with NLFs. Indeed, epoxy displays a relatively high mechanical strength, up to 90 MPa, and a stiffness of 2.5 GPa. Recent works on nanocomposites and composites reported epoxy as an effective matrix [36,37].

In this first preliminary work on the characterization and properties of TVF composites, the matrix was selected as the same DGEBA/TETA epoxy, which has been successfully considered for other Amazon native NLFs [38,39,40,41].

### 2.2. Composites Processing

The continuous and aligned TVFs with 150 mm in length were accommodated, along with the bigger dimension, inside a steel mold measured at 150 × 120 × 11.9 mm. The epoxy resin-hardener mixture was poured into the mold in a previously calculated fiber/resin ratio. For these calculations, the fiber was measured by the geometric linear density method, which consists of weighing about 100 fibers and measuring their length and diameter through an optical microscope. Due to the irregular-shaped cross-section of TVF, measurements were taken in 5 equally spaced different positions along the fiber length and repeated after a 90° rotation of the fiber [42]. The fiber density of 0.50 g/cm^3^ was obtained by dividing the weight by its calculated volume. The fiber was considered with an almost circular cross-section [43], and the volume was calculated by:

(1) $$V_{f}=\frac{\left(\pi \cdot d^{2}\right)}{4}$$

where d is the equivalent diameter measured, as described elsewhere [42]. The DGEBA/TETA epoxy resin density value of 1.11 g/cm^3^ was taken from the literature [44]. Samples were produced with 10, 20, 30 and 40 vol% fibers. Finally, after closing the mold, it was kept in a hydraulic press under pressure of 5 tons for 24 h to aid the curing process. After demolding, the composite plate was post-cured for a week at RT.

### 2.3. Thermogravimetric Analysis (TGA)

TG and DTG curves of epoxy and composites were performed on a Shimadzu machine (Tokyo, Japan). Each sample was crushed and allocated into a platinum crucible. The TGA was carried out under a nitrogen atmosphere with a heating rate of 10 °C/min in a temperature range of 25 to 700 °C. The TG/DTG analysis followed the ASTM E1131 [45].

### 2.4. Differential Scanning Calorimetry (DSC)

DSC tests were performed on a Shimadzu DSC-60 (Tokyo, Japan) calorimeter under nitrogen atmosphere with a heating rate of 10 °C/min and a temperature range of 25 to 400 °C for all samples.

### 2.5. Water Absorption

The TVF composites were subjected to the water absorption test according to ASTM D570-98 [46]. Equation (1) indicates the results of the water absorption test (%*WA*) by weight difference:

(2) $$\%WA=\frac{w_{final}-w_{initial}}{w_{initial}}$$

Based on Fick’s law, the behavior of diffusion kinetics was investigated. In polymeric materials, diffusion behavior can be classified in 3 cases, as described in previous studies [47,48,49]. In general, this classification discusses the relative mobility of the penetrant and polymer segments. These classes can be distinguished by adjusting the experimental values using the equation:

(3) $$\log\frac{M_{t}}{M_{\infty }}=\log\left(k\right)-nlog\left(t\right)$$

where $M_{t}$ is the moisture content at time *t*; $M_{\infty }$ is the moisture content at equilibrium; and *k* and *n* are constant. The theoretical model foreseen by Fick’s law was used to analyze the diffusion coefficient (*D*) in the following equation:

(4) $$\frac{M_{t}}{M_{\infty }}=\frac{4}{L}*{\left(\frac{D}{\pi }\right)}^{0.5}t^{0.5}$$

where *L* is the thickness of the samples.

### 2.6. Charpy and Izod Impact Tests

The Charpy and Izod impact tests were performed according to ASTM D6110-18 and ASTM D256-10, respectively [50,51]. The equipment used was a Pantec pendulum, model XC-50 (Sao Paulo, Brazil), with Charpy and Izod configuration, operating with a 22 J hammer. For the Charpy tests, prismatic specimens with dimensions of 127 × 12.7 × 10 mm were made with a 2.54-mm-deep notch. The tests were performed on a minimum of 9 samples for each volumetric fraction. For the Izod tests, prismatic specimens were made in the dimensions of 62.5 × 12.7 × 10 mm with a 2.54-mm-deep notch. The tests were carried out on a minimum of 12 samples for each volumetric fraction.

### 2.7. Statistical Validation

To statistically validate the level of reliability and significance of the Izod and Charpy impact results, Weibull analysis and analysis of variance (ANOVA) were performed together with the Tukey test. The Weibull parameters *β* and *θ* in the frequency distribution function are related as:

(5) $$f\left(x\right)=\exp\left[{\left(\frac{x}{\theta }\right)}^{\beta }\right]$$

These *β* and *θ* parameters, along with R^2^ accuracy, contribute to assess the level of data precision. The ANOVA and the Tukey test are able to identify differences between the average and standard deviation values of the absorbed impact energy, with a 95% confidence level.

### 2.8. Scanning Electron Microscopy (SEM)

The fractured specimens after the Izod and Charpy impact tests were analyzed using a Quanta FEG Fei Scanning Electron Microscope (FEI, Lausanne, Switzerland), operating with secondary electrons at 15–20 kV. The composite samples were gold-sputtered before SEM analysis.

## 3. Results and Discussion

### 3.1. Thermogravimetric Analysis (TGA)

**Figure 3:**
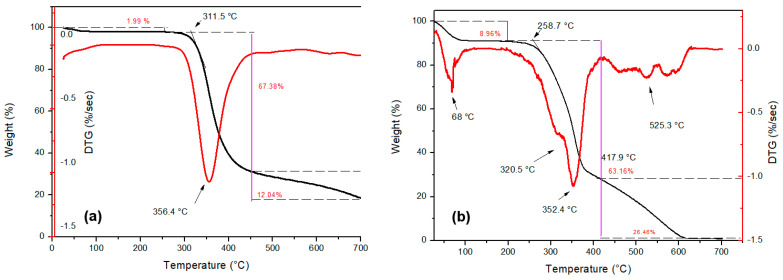
TG and DTG curves for (**a**) epoxy resin; (**b**) untreated TVFs.

Through the TGA (TG and DTG curves) it was possible to observe the process of mass loss as a function of the temperature for neat epoxy, untreated and treated fibers, as well as the TVF composites. Figure 3 shows the TG/DTG curves of DGEBA/TETA epoxy and untreated TVFs. In general, the corresponding thermograms had similar characteristics for the different investigated materials.

Figure 3a shows three stages of mass loss in the TG curves. The first occurs up to about 250 °C, with only a 1.99% loss of mass, which is probably associated with moisture desorption present in the epoxy resin. The second stage begins around 311 °C, with a maximum loss at 356 °C extending to 451 °C. This mass loss represents about 67.38% and may be associated with depolymerization and degradation of epoxy polymeric chains [52,53,54]. Finally, a third stage, with a mass loss of 12.04% up to 700 °C, is attributed to inorganic residues of the final degradation of the resin. Similar events for epoxy resin were reported by Junio et al. [52] and Silva et al. [54]. 

The TG curve for the TVF, as shown in Figure 3b, also consists of three stages, the first from 29 to 200 °C referring to water release related with moisture absorbed on the surface of a hydrophilic lignocellulosic structure, besides the evaporation of extractives associated with a 8.96% mass loss. The fastest fiber decomposition event occurs in the second stage between 258 and 417 °C, in association with the degradation of fiber structural components such as hemicellulose, cellulose and lignin [55]. Degradation for the latter occurs at a slower rate due to its complex structure [56].

The DTG curve in Figure 3b presents the thermal events more clearly. The 68 °C peak could be related to moisture loss of TVF, while the 320.5 °C “shoulder” and 352.4 °C peak are attributed to hemicellulose and cellulose decomposition, respectively [57]. At higher temperatures, above 417.9 °C, events occur that may be interconnected with lignin decomposition [58].

**Figure 4:**
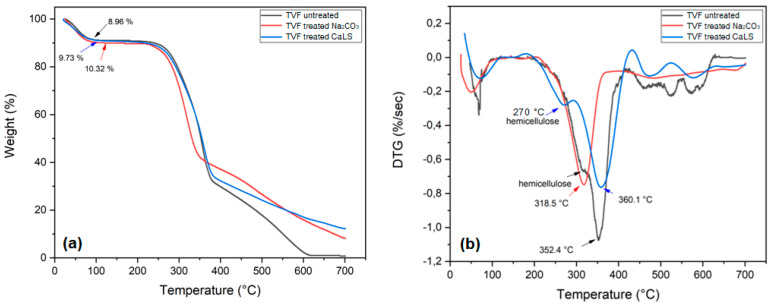
(**a**) TG curves for treated and untreated TVFs; (**b**) DTG curves for treated and untreated TVFs.

Figure 4 shows the TG and DTG curves obtained for treated fibers. In contrast to untreated fiber, Figure 4a shows that the fiber treated with Na_2_CO_3_ displayed greater affinity for moisture absorption, a fact demonstrated by its greater mass loss (10.32%) in the first stage. This same characteristic was reported by Santos et al. [27] and Fiore et al. [59] when studying natural fibers treated with sodium carbonate and sodium bicarbonate, respectively.

The pronounced “shoulder” at 270 °C for CaLS-treated fiber in Figure 4b is possibly related to hemicellulose decomposition. This same event was not observed in the fibers treated with Na_2_CO_3_, demonstrating that the treatment was efficient in removing amounts of this constituent. A DTG thermogram for CaLS-treated fibers shows a displacement of the maximum cellulose decomposition rate peaks from 352.4 to 360.1 °C, indicating an enhanced thermal stabilization. On the other hand, the alkaline treatment, for having removed much of the hemicellulose, made the cellulose more unstable and therefore, it decomposed with a higher rate at lower temperatures (318 °C). Between 400 and 580 °C for CaLS-treated fibers, it is possible to visualize peaks for lignin and lignosulfonate decomposition [35]. These peaks are not common for the fibers treated with Na_2_CO_3_, indicating that there was a possible removal of amounts of lignin from the TVFs.

**Figure 5:**
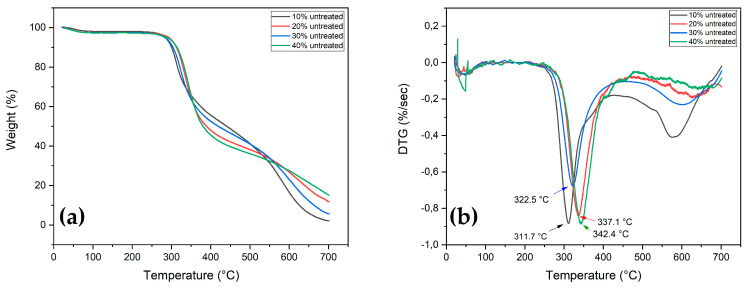
(**a**) TG curves for untreated TVF composites; (**b**) DTG curves for untreated TVF composites.

**Figure 6:**
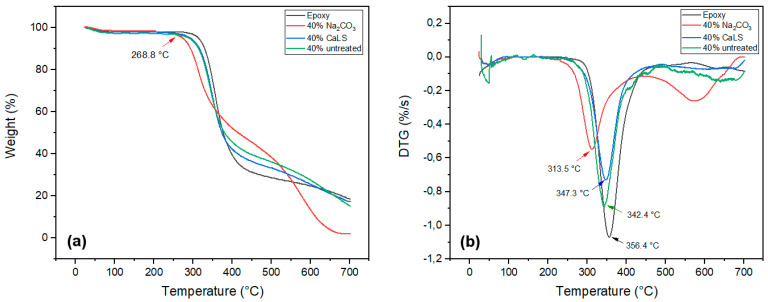
(**a**) TG curves for composites with 40 vol% of treated fibers (**b**) DTG curves for composites with 40 vol% of treated fibers.

When observing TG/DTG curves for untreated TVF composites, it can be perceived that materials tended to have greater thermal stability as more fibers were added, as shown in Figure 5a,b. This can be seen by increasing the onset decomposition temperatures and by displacing the peak of maximum decomposition of cellulose to higher temperatures. At temperatures above 400 °C, it is possible to observe peaks that might be assigned both to the degradation of polymeric chains, as well as to aromatic rings of lignin and CaLS. Figure 6 shows the TG/DTG curves for composites with 40 vol% treated TVFs.

The Figure 6 results show that Na_2_CO_3_-treated 40 vol% TVF composite is less stable than the others, starting its decomposition process at 268.8 °C. This fact is recurrent, since it has already been observed in the TVF thermogram after treatment, as shown in Figure 4b. The low properties evidenced for TVFs treated with Na_2_CO_3_ and their composites might be associated with the non-transformation of cellulose I into cellulose II. The transition from cellulose I to cellulose II depends on the interaction of two factors: NaOH concentration and treatment temperature [60]. This phenomenon plays a fundamental role in the thermal property since cellulose II has more intermolecular hydrogen bonds than cellulose I, favoring increased thermal stability [61]. Previous studies have reported a similar effect on sisal [59] and coir [62] fibers treated at RT with NaHCO_3_ and NaOH, respectively. Additionally, Pinheiro et al. [63] and Barreto et al. [64] verified the improvement of thermal stability after alkaline treatment on other NLFs due to the cellulose II formation. 

Table 1 presents the main TGA parameters obtained for epoxy, untreated and treated TVFs and the composites investigated. The beginning of thermal decomposition (T_onset_) for all materials was obtained by the tangent method.

The results in Table 1 indicate that the composites presented better thermal stability in relation to isolated TVFs. The increase in the percentage of fibers led to an increase in the working temperature of the composites, retarding the main weight loss of the composite, as shown in Figure 5. However, these temperatures for all samples are slightly lower when compared to neat epoxy. These results corroborate previous studies in which natural fibers were used as epoxy matrix reinforcement [65,66]. Based on these results, a thermal stability limit of 296.3 °C can be established for untreated 40 vol% TVF composites and 268.8 °C for Na_2_CO_3_-treated 40 vol% TVF composites.

### 3.2. Differential Scanning Calorimetry (DSC)

**Figure 7:**
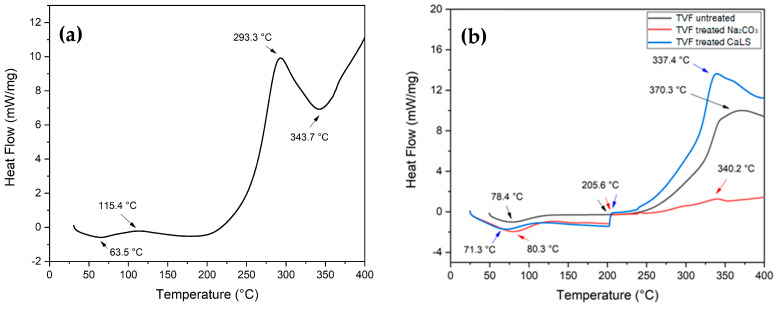
DSC curves for (**a**) neat epoxy resin; (**b**) untreated and treated TVFs.

Figure 7 shows the DSC curves for the investigated materials. In particular, Figure 7a reveals an endothermic peak at 63.5 °C, which is associated with glass transition (T_g_) and a loss of moisture from the epoxy resin [66,67,68,69]. In addition, two exothermic events are noticeable. The first occurs at 115.4 °C and is probably linked to the main resin curing process. The second exothermic event occurs at 293.3 °C and can be attributed to cross bonds such as homopolymerization and esterification of epoxy groups [70]. Finally, a last endothermic peak appears at 343.7 °C, which characterizes the possible degradation and rupture of the polymeric chains [70,71], corroborating the results of TG in Figure 3.

**Figure 8:**
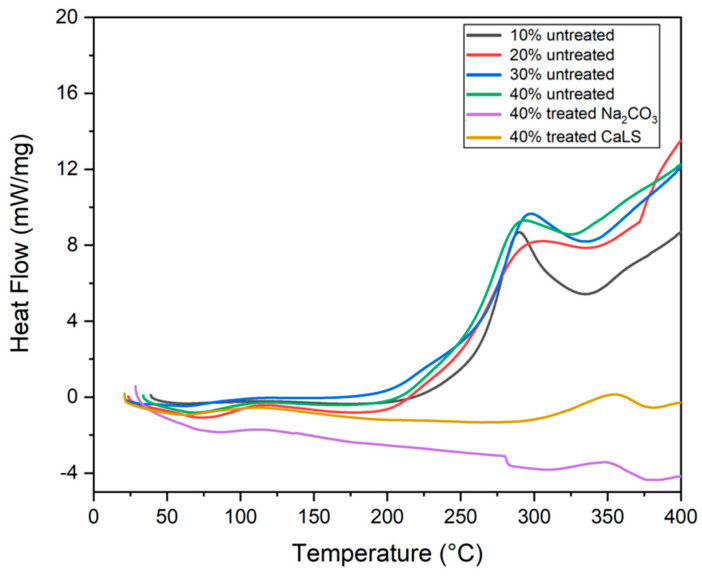
DSC curves for untreated and treated TVF composites.

The DSC curve for untreated and treated TVF, shown in Figure 7b, initially presents endothermic peaks between 70–80 °C that refer to moisture loss and TVF T_g_. The T_g_ found for TVF in this work was very similar to natural fibers previously studied, such as carnauba (107 °C) [52], caranan (64 °C) [66], PALF (75 °C) [72] and jute (61 °C) [73]. Although these events are slightly different from those approached by TG, they fall within the same temperature range. The following events of thermal degradation, which begins for all fiber samples around 205.6 °C and extends up to 370.3 °C for the untreated fiber, are probably linked to the onset of decomposition of hemicellulose, cellulose and lignin, which take more time to start their degradation. As the test occurred up to 400 °C, there are no more events that could be related to the degradation of lignin and lignosulfonate above this plateau. Figure 8 shows the DSC curves for composites made with TVF with and without treatment.

Events are a combination of previously results seen for epoxy and isolated fibers, as shown Figure 8. In addition, it can be is observed that the T_g_ of the composites, as well as the moisture loss event, varied between 63–85 °C, as also presented in the TG curves of Figure 6a. Around 115 °C is the probable start of the curing process of the polymeric matrix. Exothermic and endothermic peaks are present between 290–350 °C; these are probably related to epoxy group crosslinks, decomposition of the main fibers’ constituents and degradation of the polymer chains.

### 3.3. Water Absorption

Water absorption of neat epoxy and TVF-incorporated epoxy composites with both untreated and treated fibers can be seen in Figure S1. It can be observed in this figure that the absorption of water by the composites increases with the time of immersion and the fiber content. This characteristic was already expected as the fibers are hydrophilic materials [70].

**Figure 9:**
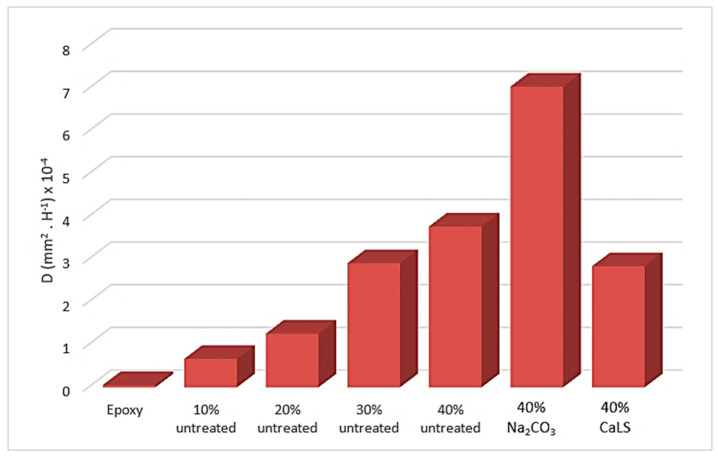
Diffusion coefficient values for composite samples.

The treatments performed in the fibers showed two distinct behaviors in the 40 vol% samples. The fiber composites treated with Na_2_CO_3_ absorbed 19% more water than those with untreated fibers. This is probably due to the fact that the alkaline treatment promoted a cleaning on the fiber surface, making it more exposed to retain water. In addition, porosity and microvoids present in samples from processing may have also contributed to absorb water. On the other hand, treatment with CaLS reduced water absorption by 8% compared to untreated composites. This may indicate a slight improvement in the fiber/matrix interfacial bond as well as the treatment efficiency in the stability and durability of composites [33,74,75]. Figure 9 and Table 2 show that D values from Equation (3) increase as more fibers are added to the matrix. This reveals that composites with higher TVF contents have a greater ability to penetrate water molecules through the fiber/matrix interface and, therefore, develop a lower moisture resistance. The high D value verified for Na_2_CO_3_-treated TVF composites indicated it to be the most susceptible to water molecule penetration. In contrast, the CaLS decreased the D value, showing that water molecules have less ability to move within the material.

As presented in Table 2, the lower values of parameter k in Equation (2), due to the increased proportion of fibers, are associated with a weak affinity between water and composite. This reveals that there was good protection of the fibers by the matrix and that the impregnation of the fiber by the epoxy was not impaired by the increase in TVF content.

### 3.4. Statistical Validation of the Charpy and Izod Impact Tests

Table 3 shows the Weibull parameters (β and R^2^) as well as the characteristic absorbed energy θ. The θ values are similar to those found for the mean absorption energy (E_absorbed_) obtained in the Charpy and Izod tests. 

Another point that becomes more noticeable when analyzing Table 3 is the relatively high dispersion for 40 vol% samples, which is associated with heterogeneity of natural fibers [76]. This dispersion may imply a possible statistical similarity between the composites of both groups of fibers, without and with treatment. To confirm this hypothesis, the ANOVA statistical analysis was performed for the obtained results.

Tables S1 and S2 present the ANOVA of the results of Charpy and Izod impact resistance for untreated 40 vol% TVF composites, respectively. The data in Table S1 indicate that the hypothesis of equality between the values of absorbed energy Charpy and Izod for groups 0–40 vol% of untreated TVF is rejected with a 95% confidence level, because F_cal_ = 12.60 and 27.43 are higher than F_critical_ = 2.64 and 2.57.

Table S2 shows that the treatments with Na_2_CO_3_ and CaLS did not present a direct influence on the absorption of impact energy in both tests for the 40 vol% samples. This is justified by comparing the values of F_cal_ = 1.26 and 1.12 with F_critical_ = 3.47 and 3.28, obtaining F_cal_ < F_critical_. Therefore, with 95% confidence, the hypothesis of equity among treatment averages is assumed.

Despite the non-significant increase in property, none of the treatments showed a marked decrease in resistance to impact in the groups analyzed in Figures S1 and S2 as well as in Table S2, available in the Supplementary Materials. However, the 40 vol% TVF treated with CaLS not only showed a higher Charpy absorbed energy than the plain epoxy but also a higher value than any other untreated TVF composite listed in Table 3. Furthermore, the 40 vol% TVF treated with Na_2_CO_3_ displayed a higher Izod absorbed energy than any other untreated TVF composite. These promising preliminary results indicated that both TVF treatments might have been effective in improving the impact resistance of epoxy composites reinforced with more than 30 vol% TVFs. Ongoing research work is extending the percentage of CaLS and Na_2_CO_3_ as well as the volume fractions of incorporation of TVF.

To verify which volume fraction of untreated TVF showed better a Charpy and Izod impact resistance on composites, the Tukey test was applied to compare individual performance with a 95% confidence level. 

The HSD calculated for the Charpy test was 10.22 J/m, and the differences above this value are considered significant. The values in Table S3 show that the Charpy impact resistance for the 40 vol% TVF composite was in fact higher than those of the other composites. However, a decrease in the absorption energy was observed for the composite with 20 vol% compared to the neat epoxy, which suggests that the TVF acted only as filler in this volume fraction. Part of this decrease in energy may be associated with low fiber/matrix compatibility, which is often due to the presence of moisture on the surface of natural fibers and can lead to crack initiation, reducing the impact resistance of the composite. Furthermore, these results indicate that only 40 vol% of TVF might act as an effective reinforcement for epoxy resin.

For the Izod test, the calculated HSD was 10.78 J/m. Table S4 revealed that the Izod impact resistance of the composite with 40 vol% TVF confirmed its better performance. On the other hand, the Tukey test pointed to decreased impact resistance for composites with 10 and 20 vol% TVFs in relation to neat epoxy. Some reasons can be cited for this fact, such as the weak fiber/matrix adhesion and pressing with excessive load during plate processing, which may have generated micro-cracks in the material.

### 3.5. Scanning Electron Microscopy Analysis

**Figure 10:**
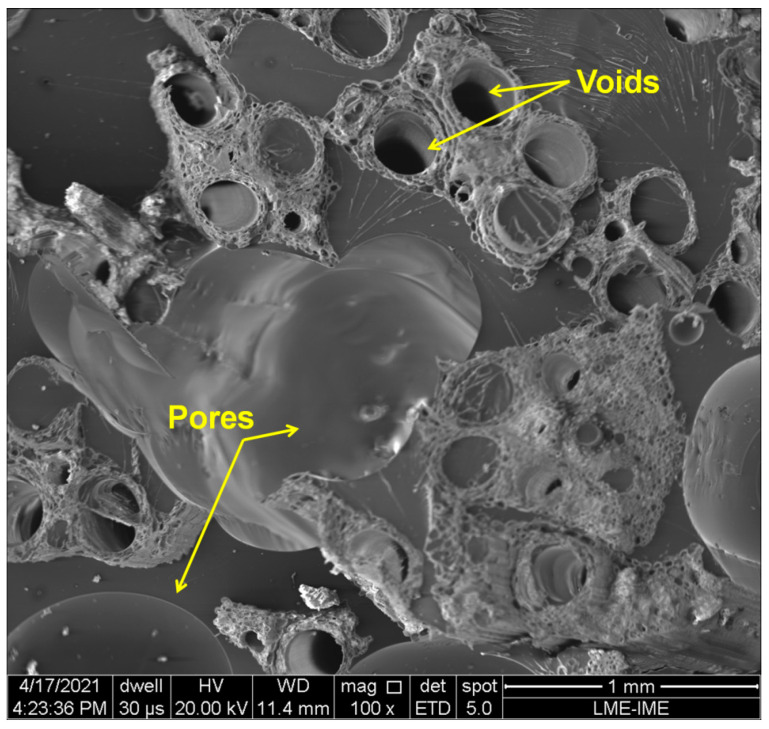
SEM images of cross-sections of untreated 30 vol% TVF composite.

SEM image of the typical cross-section of untreated 30 vol% TVF composites is shown in Figure 10. In this figure, the many voids of TVF as well as the presence of pores resulting from the manufacturing process of composites, which can impair their mechanical properties, can be noted.

**Figure 11:**
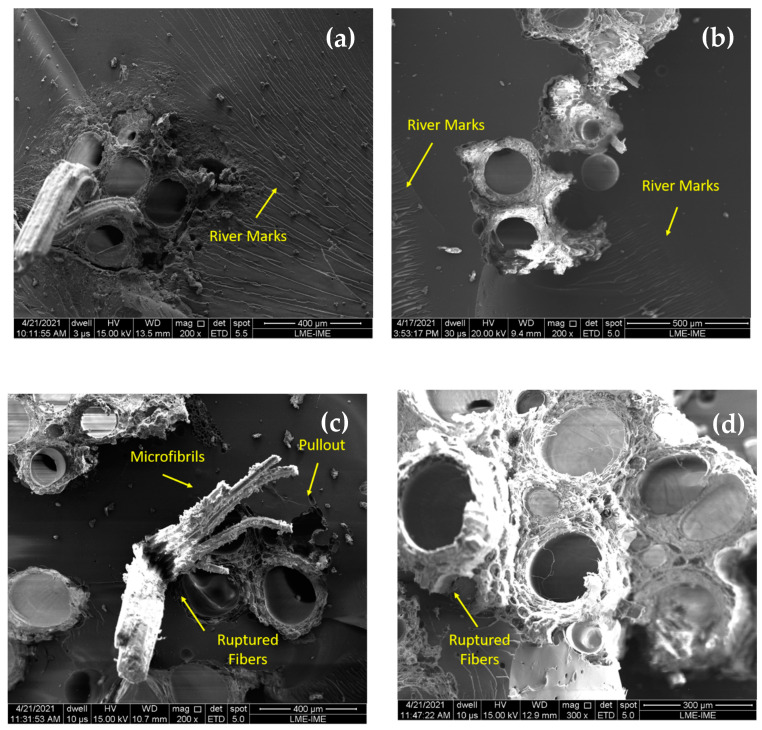
SEM of fracture surfaces of composites incorporated with untreated TVFs after Charpy impact tests: (**a**) 10 vol% TVF; (**b**) 20 vol% TVF; (**c**) 30 vol% TVF; and (**d**) 40 vol% TVF.

**Figure 12:**
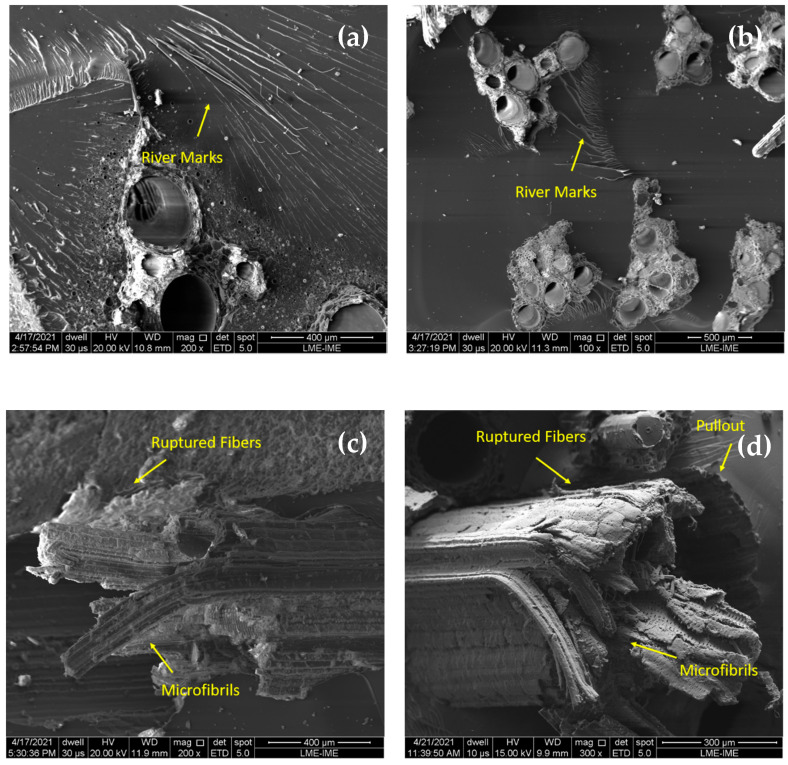
SEM of fracture surfaces of composites incorporated with untreated TVFs after Izod impact tests: (**a**) 10 vol% TVF; (**b**) 20 vol% TVF; (**c**) 30 vol% TVF; and (**d**) 40 vol% TVF.

The increase in impact resistance with the increase in the volume fraction of TVF fibers in the composite might be assigned to the fracture mechanisms acting in the composites. In order to confirm and better understand the evolution of fracture mechanisms acting on the materials tested, Figure 11 and Figure 12 show the fracture surfaces of the samples’ composites with untreated TVF of the Charpy and Izod tests, respectively.

Figure 11a,b and Figure 12a,b reveal a rather brittle epoxy matrix fracture, due to the presence of “river marks” on the impact surface of the samples. In addition, in these composites it was possible to observe that the crack spreads catastrophically, inferring that there was no effective reinforcement. According to Figure S3 and Table 3, the composites with 30 and 40 vol% of TVFs obtained the most efficient performance of the fibers due to higher energy absorption. In these composites, more complex fracture mechanisms were activated, such as fiber rupture and pullout, as shown in Figure 11c,d and Figure 12c,d. In these figures, the fracture mechanisms that occurred are associated with the rupture of microfibrils that provide an additional free surface and have the ability to increase the energy of the absorbed impact. This same effect was reported by Reis et al. [39] when evaluating the energy of impact absorbed by guaruman fiber-reinforced epoxy matrix composites.

**Figure 13:**
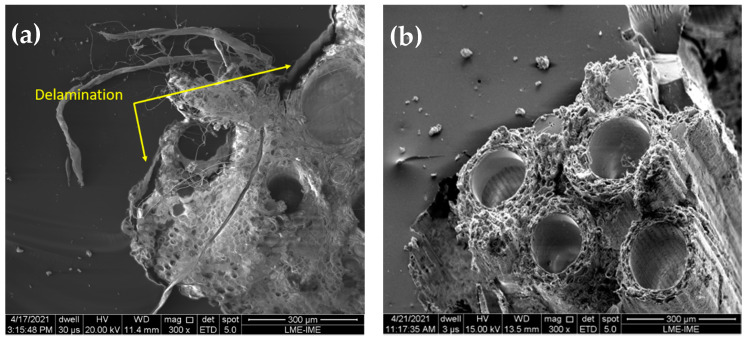
SEM of fracture surface of composites incorporated with untreated TVFs after Izod impact test: (**a**) 30 vol% TVF; and (**b**) 40 vol% TVF.

Another relevant characteristic to note is the delamination between the fiber and the matrix, as observed in Figure 13a.

It is important noticing that TVF has a large number of pores and voids in Figure 13b. This large number of pores and voids is likely to be related to the relatively lower density of TVF as compared to NFLs [77]. This fact makes TVF quite advantageous as compared to several other NLFs for use as reinforcement for polymer matrix composites. Indeed, in many end applications, parts with excellent specific mechanical properties are desired, for example in the automotive and aeronautical industry.

### 3.6. Recommendation for Future Research

XPS is an effective way to verify the fiber surface condition before and after treatment as well as the amount of its functionalization. As an example, Revol et al. [78] investigated the functionalization of cellulose-based fiber (Viscord^TM^) with Na_2_CO_3_ (0.1 wt%) for 30 min at RT, with 1 g of fiber/20 mL of solution. They observed that the number of C–C groups, which indicates the content of impurities present on the fiber surface, did not change between untreated and after Na_2_CO_3_ treated fiber. However, the O/C ratio, associated with the number of hydroxyl groups available on the surface, increased from 53 to 61%. It is worth mentioning that COOR groups are of interest when the functionalization of the surface is taken into account. The authors also verified a large proportion of the COOR (73.5% of C–O–H groups) after Na_2_CO_3_ treatment, but this value barely changed in comparison to the untreated fiber, and impurities remained present on the fiber surface [78]. Therefore, these results indicate that the Na_2_CO_3_ might not be effective to functionalize a natural fiber. In this way, the effect of different Na_2_CO_3_ treatment conditions on the functionalization of the TVF surface will be further investigated in future studies.

## 4. Conclusions

For the first time, the thermal, water absorption and mechanical properties of epoxy matrix composites incorporated with Amazon native titica vine fibers (TVFs) treated with both sodium carbonate (Na_2_CO_3_) and calcium lignosulfonate (CaLS) were investigated.

The results of TGA/DTG and DSC thermal analysis indicate that, in spite of lower thermal stability than that of neat epoxy, there was an increase in the composites’ thermal stability as the TVFs were added. The composite with 40 vol% of untreated TVFs stood out from the others, with an initial decomposition temperature around 296 °C. The DSC presented curves of events that corroborated the DTG, as well as peaks associated with the glass transition of the composites ranging from 60–85 °C and the beginning of the curing process of the polymeric matrix around 115 °C.

The water absorption test in the composites showed that the samples with higher incorporation percentages absorbed more water, a fact that was expected due to the hydrophilic characteristics of natural lignocellulosic fibers. Treatment with Na_2_CO_3_ increased the water absorption of the composites with 40 vol% TVF by 19% compared to untreated composites, while treatment with CaLS decreased water absorption by 8%.

Mechanical testing by impact Charpy and Izod showed that up to 30 vol% of fiber fraction resulted in similar or lower impact resistance than the neat epoxy. However, composites reinforced with 40 vol% CaLS-treated TVF showed a 31% improvement in Charpy tests, while Na_2_CO_3_-treated 40 vol% TVF displayed a 45% improved Izod impact resistance, in relation to neat epoxy. Statistical validation by ANOVA and Tukey test confirmed that the TVF treatments in 40 vol% composites are associated with the highest impact resistances. It also revealed that 40 vol% TVF is a threshold limit for improved impact resistance.

Through SEM fractographies, it was possible to evaluate the main energy absorption mechanisms present in untreated fiber composites. The samples with smaller incorporation fractions, 10 and 20 vol%, showed brittle characteristics. On the other hand, for the largest volumetric fraction of fibers, 30 and 40 vol% TVF, the presence of mechanisms such as pullout and fiber rupture, as well as delamination, which are directly associated with high absorption of Charpy and Izod impact energy, was observed.

The results indicate that TVF-incorporated composites are viable for engineering applications in view of their lightness and high working temperature, as well as the positive effect of CaLS treatment on less water absorption and increased impact energy absorbed with the addition of fibers. These characteristics are interesting for aerospace, ballistics and automobile industries, especially when considering the low density required in the project. In particular, the relative cost effectiveness together with easy processing and thermal stability suggest a possible use of TVF epoxy composites in food packing.

## Figures and Tables

**Table 1:** Thermogravimetric parameters for neat epoxy, untreated and treated titica vine fiber (TVF) and their composites.

Sample	Mass Loss up to 200 °C (%)	Initial Decomposition Temperature (°C)	Temperature of Maximum Decomposition Rate (°C)	Mass Loss at the End of Second Stage (%)	Mass Loss at 700 °C (%)
Epoxy	1.99	311.5	356.4	67.38	81.41
TVF untreated	8.96	258.7	352.4	63.16	98.58
TVF Na_2_CO_3_	10.32	249.8	318.5	54.11	91.79
TVF CaLS	9.73	257.0	360.1	56.24	87.78
10% untreated	1.95	289.1	311.7	53.22	97.86
20% untreated	2.57	294.5	337.1	54.24	87.97
30% untreated	2.61	292.1	322.5	51.12	94.19
40% untreated	2.83	296.3	342.4	60.41	84.65
40% Na_2_CO_3_	1.67	268.8	313.5	54.06	98.07
40% CaLS	2.11	293.7	347.3	60.33	82.67

**Table 2:** Water absorption for untreated and treated TVF composites. Diffusion coefficient D and parameter k.

Sample	D (mm^2^·h^−1^) × 10^−4^	k
Epoxy	0.054	0.996
10% untreated	0.670	0.989
20% untreated	1.260	0.984
30% untreated	2.910	0.973
40% untreated	3.770	0.975
40% treated Na_2_CO_3_	7.050	0.966
40% treated CaLS	2.840	0.974

**Table 3:** Weibull parameters for resistance to Impact Charpy and Izod of untreated and treated TV fibers composites.

	Charpy					Izod				
Sample	β	θ	R^2^	E_absorbed_ (J/m)	Standard Deviation	β	θ	R^2^	E_absorbed_ (J/m)	Standard Deviation
Epoxy	6.86	61.41	0.98	61.52	7.89	3.03	46.31	0.83	42.24	15.91
10% untreated	6.7	69.25	0.94	66.44	8.72	2.23	23.29	0.96	24.20	6.10
20% untreated	8.45	51.78	0.98	50.54	5.73	2.27	23.6	0.96	25.43	6.58
30% untreated	10.23	62.24	0.98	62.13	5.22	10.27	42.67	0.82	41.01	4.37
40% untreated	5.8	74.67	0.96	75.05	7.32	4.99	61.02	0.92	58.65	9.26
40% treated Na_2_CO_3_	7.2	71.19	0.93	68.28	8.08	4.34	67.07	0.79	61.11	15.20
40% treated CaLS	3.4	88.5	0.94	80.59	24.53	4.27	59.52	0.91	53.31	13.89

## Data Availability

The data presented in this study are available on request from the corresponding author.

## Supplementary Materials

The following are available online at https://www.mdpi.com/article/10.3390/polym13234079/s1, Figure S1: Water absorption curve for neat epoxy as well as untreated and treated TVF composites, Figure S2: Variation of the absorbed impact energy of the composites in relation to the volume fraction of untreated TVF for (a) Charpy; and (b) Izod tests, Figure S3: Variation of the absorbed impact energy of the composites in relation to the volumetric fraction of untreated and treated TVF for the (a) Charpy; (b) Izod, Table S1: ANOVA for the absorbed impact energy Charpy and Izod of composites with different volume fractions of untreated TVFs, Table S2: ANOVA for the absorbed impact energy Charpy and Izod of composites with 40 vol% of untreated and treated TVFs, Table S3: HSD as measured by Tukey test for the Charpy absorbed impact energy of composites with volume different fractions of untreated TVFs, Table S4: HSD as measured by Tukey test for the Izod absorbed impact energy of composites with volume different fractions of untreated TVFs.

## References

[1] Mohammed L., Ansari M.N.M., Pua G., Jawaid M., Islam S. (2015). A review on natural fiber reinforced polymer composite and its applications. Int. J. Polym. Sci..

[2] May-Pat A., Valadez-González A., Herrera-Franco P.J. (2013). Effect of fiber surface treatments on the essential work of fracture of HDPE-continuous henequen fiber-reinforced composites. Polym. Test..

[3] Luz F.S., Garcia Filho F.C., Del-Rio M.T.C., Nascimento L.F.C., Pinehro W.A., Monteiro S.N. (2020). Graphene-incorporated natural fiber polymer composites: A first overview. Polymers.

[4] Zhang Z., Cai S., Li Y., Wang Z., Long Y., Yu T., Shen Y. (2020). High performances of plant fiber reinforced composites—A new insight from hierarchical microstructures. Compos. Sci. Technol..

[5] Vigneshwaran S., Sundarakannan R., John K., Johnson R.D.J., Prasath K.A., Ajith S., Arumugaprabu V., Uthayakumar M. (2020). Recent advancement in the natural fiber polymer composites: A comprehensive review. J. Clean. Prod..

[6] Karimah A., Ridho M.R., Munawar S.S., Adi D.S., Damayanti R., Subiyanto B., Fatriasari W., Fudholi A. (2021). A review on natural fibers for development of eco-friendly bio-composite: Characteristics, and utilizations. J. Mater. Res. Technol..

[7] Potluri R., Krishna N.C. (2020). Potential and applications of green composites in industrial space. Mater. Today Proc..

[8] Kumar R., Haq M.I.U., Raina A., Anand A. (2018). Industrial applications of natural fibre-reinforced polymer composites—Challenges and opportunities. Int. J. Sustain. Eng..

[9] Dunne R., Desai D., Sadiku R., Jayaramudu J. (2016). A review of natural fibres, their sustainability and automotive applications. J. Reinf. Plast. Compos..

[10] Reddy B.M., Reddy Y.V.M., Reddy B.C.M., Reddy R.M. (2020). Mechanical, morphological, and thermogravimetric analysis of alkali-treated Cordia-Dichotoma natural fiber composites. J. Nat. Fibers.

[11] Li M., Pu Y., Thomas V.M., Yoo C.G., Ozcan S., Deng Y., Nelson K., Ragauskas A.J. (2020). Recent advancements of plant-based natural fiber–reinforced composites and their applications. Compos. Part B Eng..

[12] Dixit S., Goel R., Dubey A., Shivhare P.R., Bhalavi T. (2017). Natural fibre reinforced polymer composite materials—A review. Polym. Renew. Resour..

[13] Kalia S., Kaith B.S., Kaurs I. (2011). Cellulose Fibers: Bio and Nano Polymer Composites: Green Chemistry and Technology.

[14] Poletto M., Júnior H.L.O., Zattera A.J. (2015). Thermal decomposition of natural fibers: Kinetics and degradation mechanisms. Reactions and Mechanisms in Thermal Analysis of Advanced Materials.

[15] Sanjay M.R., Madhu P., Jawaid M., Senthamaraikannan P., Senthil S., Pradeep S. (2018). Characterization and properties of natural fiber polymer composites: A comprehensive review. J. Clean. Prod..

[16] Pickering K.L., Efendy M.G.A., Le T.M. (2016). A review of recent developments in natural fibre composites and their mechanical performance. Compos. Part A Appl. Sci. Manuf..

[17] Kabir M.M., Wang H., Lau K.T., Cardona F. (2012). Chemical treatments on plant-based natural fibre reinforced polymer composites: An overview. Compos. Part B Eng..

[18] Al-Maharma A.Y., Al-Huniti N. (2019). Critical review of the parameters affecting the effectiveness of moisture absorption treatments used for natural composites. J. Compos. Sci..

[19] Faruk O., Bledzki A.K., Fink H.-P., Sain M. (2012). Biocomposites reinforced with natural fibers: 2000–2010. Prog. Polym. Sci..

[20] Gholampour A., Ozbakkaloglu T. (2019). A review of natural fiber composites: Properties, modification and processing techniques, characterization, applications. J. Mater. Sci..

[21] Krishnudu D.M., Sreeramulu D., Reddy P.V. (2020). Alkali treatment effect: Mechanical, thermal, morphological, and spectroscopy studies on abutilon indicum fiber-reinforced composites. J. Nat. Fibers.

[22] Serra-Parareda F., Espinach F.X., Pelach M.À., Méndez J.A., Vilaseca F., Tarrés Q. (2020). Effect of NaOH treatment on the flexural modulus of hemp core reinforced composites and on the intrinsic flexural moduli of the fibers. Polymers.

[23] de Araujo Alves Lima R., Cavalcanti D.K., de Souza e Silva Neto J., da Costa H.M., Banea M.D. (2020). Effect of surface treatments on interfacial properties of natural intralaminar hybrid composites. Polym. Compos..

[24] Bartos A., Utomo B.P., Kanyar B., Anggono J., Soetaredjo F.E., Móczó J., Pukánszky B. (2020). Reinforcement of polypropylene with alkali-treated sugarcane bagasse fibers: Mechanism and consequences. Compos. Sci. Technol..

[25] Raia R.Z., Iwakiri S., Trianoski R., de Andrade A.S., Kowalski E.L. (2021). Effects of alkali treatment on modification of the Pinus fibers. Matéria.

[26] Machaka M., Basha H., Abou Chakra H., Elkordi A. (2014). The effect of using fan palm natural fibers on the mechanical properties and durability of concrete. Int. J. Mater. Sci. Eng..

[27] dos Santos J.C., Siqueira R.L., Vieira L.M.G., Freire R.T.S., Mano V., Panzera T.H. (2018). Effects of sodium carbonate on the performance of epoxy and polyester coir-reinforced composites. Polym. Test..

[28] Megiatto J.D., Cerrutti B.M., Frollini E. (2016). Sodium lignosulfonate as a renewable stabilizing agent for aqueous alumina suspensions. Int. J. Biol. Macromol..

[29] Ye D.Z., Jiang L., Hu X.-Q., Zhang M.-H., Zhang X. (2016). Lignosulfonate as reinforcement in polyvinyl alcohol film: Mechanical properties and interaction analysis. Int. J. Biol. Macromol..

[30] Klauberg C., Vidal E., Silva C.A., Bentes M.D.M., Hudak A.T. (2016). Sampling methods for titica vine (Heteropsis spp.) inventory in a tropical forest. Ann. For. Sci..

[31] Costa U.O., Nascimento L.F.C., Garcia J.M., Bezerra W.B.A., da Costa G.F.F., da Luz F.S., Pinheiro W.A., Monteiro S.N. (2020). Mechanical properties of composites with graphene oxide functionalization of either epoxy matrix or curaua fiber reinforcement. J. Mater. Res. Technol..

[32] Demosthenes L.C.C., Nascimento L.F.C., Monteiro S.N., Costa U.O., Garcia Filho F.C., Luz F.S., Oliveira M.S., Ramos F.J.H.T.V., Pereira A.C., Braga F.O. (2020). Thermal and structural characterization of buriti fibers and their relevance in fabric reinforced composites. J. Mater. Res. Technol..

[33] Cunha J.S.C., Neto H.E.O., Giacon V.M., Manzato L., Silva C.G. (2021). Study on mechanical and thermal properties of Amazon fibers on the polymeric biocomposites: Malva and tucum. Fibers Polym..

[34] Reis R.H.M., Nunes L.F., da Luz F.S., Candido V.S., da Silva A.C.R., Monteiro S.N. (2021). Ballistic performance of guaruman fiber composites in multilayered armor system and as single target. Polymers.

[35] Oliveira F., Silva C.G., Ramos L.A., Frollini E. (2017). Phenolic and lignosulfonate-based matrices reinforced with untreated and lignosulfonate-treated sisal fibers. Ind. Crop. Prod..

[36] Kerche E.F., da Silva V.D., Fonseca E., Salles N.A., Schrekker H.S., Amico S.C. (2021). Epoxy-based composites reinforced with imidazolium ionic liquid-treated aramid pulp. Polymers.

[37] Jojibabu P., Zhang Y.X., Prusty B.G. (2020). A review of research advances in epoxy-based nanocomposites as adhesive materials. Int. J. Adhes. Adhes..

[38] Nascimento L.F.C., Louro L.H.L., Monteiro S.N., Lima É.P., da Luz F.S. (2017). Mallow fiber-reinforced epoxy composites in multilayered armor for personal ballistic protection. JOM.

[39] Reis R.H., Filho F.C.G., Nunes L.F., Candido V.S., Silva A.C., Monteiro S.N. (2021). Impact resistance of epoxy composites reinforced with Amazon guaruman fiber: A Brief Report. Polymers.

[40] Costa U.O., Nascimento L.F.C., Bezerra W.B.A., Aguiar V.O., Pereira A.C., Monteiro S.N., Pinheiro W.A. (2021). Dynamic mechanical behavior of graphene oxide functionalized curaua fiber-reinforced epoxy composites: A brief report. Polymers.

[41] Oliveira M.S., Luz F.S.D., Souza A.T., Demosthenes L.C.D.C., Pereira A.C., Braga F.D.O., Figueiredo A.B.-H.S., Monteiro S.N. (2020). Tucum fiber from Amazon *Astrocaryum vulgare* palm tree: Novel reinforcement for polymer composites. Polymers.

[42] Da Luz F.S., Paciornik S., Monteiro S.N., Da Silva L.C., Tommasini F.J., Cândido V.S. (2017). Porosity assessment for different diameters of coir lignocellulosic fibers. JOM.

[43] Truong M., Zhong W., Boyko S., Alcock M. (2009). A comparative study on natural fibre density measurement. J. Text. Inst..

[44] Ribeiro M.P., Neuba L.M., Silveira P.H.P.M., Luz F.S., Figueiredo A.B.H.S., Monteiro S.N., Moreira M.O. (2021). Mechanical, thermal and ballistic performance of epoxy composites reinforced with Cannabis sativa hemp fabric. J. Mater. Res. Technol..

[45] American Society for Testing and Materials (2003). E 1131: Standard Test Method for Compositional Analysis by Thermogravimetry.

[46] American Society for Testing and Materials (2018). D570-98: Standard Test Method for Water Absorption of Plastics.

[47] Espert A., Vilaplana F., Karlsson S. (2004). Comparison of water absorption in natural cellulosic fibres from wood and one-year crops in polypropylene composites and its influence on their mechanical properties. Compos. Part A Appl. Sci. Manuf..

[48] Megiatto J.D., Oliveira F.B., Rosa D.S., Gardrat C., Castellan A., Frollini E. (2007). Renewable resources as reinforcement of polymeric matrices: Composites Based on Phenolic Thermosets and Chemically Modified Sisal Fibers. Macromol. Biosci..

[49] Osman E.A., Vakhguelt A., Sbarski I., Mutasher S.A. (2012). Kenaf/recycled jute natural fibers unsaturated polyester composites: Water absorption/dimensional stability and mechanical properties. Int. J. Comput. Mater. Sci. Eng..

[50] American Society for Testing and Materials (2018). D6110-18: Standard Test Method for Determining the Charpy Impact Resistance of Notched Specimens of Plastics.

[51] American Society for Testing and Materials (2000). D256-10: Standard Test Methods for Determining the Izod Pendulum Impact Resistance of Plastics.

[52] Junio R.F.P., Nascimento L.F.C., Neuba L.M., Souza A.T., Moura J.V.B., Filho F.C.G., Monteiro S.N. (2020). *Copernicia Prunifera* leaf fiber: A promising new reinforcement for epoxy composites. Polymers.

[53] Silva T.T.D., Silveira P.H.P.M.D., Ribeiro M.P., Lemos M.F., Silva A.P., Monteiro S.N., Nascimento L.F.C. (2021). Thermal and chemical characterization of kenaf fiber (*Hibiscus cannabinus*) reinforced epoxy matrix composites. Polymers.

[54] Jin F.-L., Park S.-J. (2012). Thermal properties of epoxy resin/filler hybrid composites. Polym. Degrad. Stab..

[55] Monteiro S.N., Calado V., Margem F.M., Rodriguez R.J. (2012). Thermogravimetric stability behavior of less common lignocellulosic fibers—A review. J. Mater. Res. Technol..

[56] Yang H., Yan R., Chen H., Lee D.H., Zheng C. (2007). Characteristics of hemicellulose, cellulose and lignin pyrolysis. Fuel.

[57] Xia L., Zhang C., Wang Y., Xu W. (2020). Morphologies and properties of Juncus effusus fiber after alkali treatment. Celluloes.

[58] Sgriccia N., Hawley M. (2007). Thermal, morphological, and electrical characterization of microwave processed natural fiber composites. Compos. Sci. Technol..

[59] Fiore V., Scalici T., Nicoletti F., Vitale G., Prestipino M., Valenza A. (2016). A new eco-friendly chemical treatment of natural fibres: Effect of sodium bicarbonate on properties of sisal fibre and its epoxy composites. Compos. Part B Eng..

[60] Liu Y., Hu H. (2008). X-ray diffraction study of bamboo fibers treated with NaOH. Fibers Polym..

[61] El Oudiani A., Chaabouni Y., Msahli S., Sakli F. (2011). Crystal transition from cellulose I to cellulose II in NaOH treated *Agave americana* L. fibre. Carbohydr. Polym..

[62] Silva G.G., De Souza D.A., Machado J.C., Hourston D.J. (2000). Mechanical and thermal characterization of native brazilian coir fiber. J. Appl. Polym. Sci..

[63] Pinheiro I.F., Morales A.R., Mei L.H.I. (2014). Polymeric biocomposites of poly (butylene adipate-co-terephthalate) reinforced with natural Munguba fibers. Cellulose.

[64] Barreto A.C.H., Rosa D.S., Fechine P.B.A., Mazzetto S.E. (2011). Properties of sisal fibers treated by alkali solution and their application into cardanol-based biocomposites. Compos. Part A Appl. Sci. Manuf..

[65] Neuba L.M., Junio R.F.P., Ribeiro M.P., Souza A.T., Lima E.S., Filho F.C.G., Figueiredo A.B.H.S., Braga F.O., Azevedo A.R.G., Monteiro S.N. (2020). Promising mechanical, thermal, and ballistic properties of novel epoxy composites reinforced with *Cyperus malaccensis* sedge fiber. Polymers.

[66] Souza A.T., Junio R.F.P., Neuba L.M., Candido V.S., Silva A.C.R., Azevedo A.R.G., Monteiro S.N., Nascimento L.F.C. (2020). Caranan fiber from *Mauritiella armata* palm tree as novel reinforcement for epoxy composites. Polymers.

[67] Jesuarockiam N., Jawaid M., Zainudin E.S., Sultan M.T.H.S., Yahaya R. (2019). Enhanced thermal and dynamic mechanical properties of synthetic/natural hybrid composites with graphene nanoplateletes. Polymers.

[68] Naveen J., Jawaid M., Zainudin E.S., Sultan M.T., Yahaya R., Majid M.A. (2019). Thermal degradation and viscoelastic properties of Kevlar/Cocos nucifera sheath reinforced epoxy hybrid composites. Compos. Struct..

[69] Revanth J.S., Madhav V.S., Sai Y.K., Krishna D.V., Srividya K., Sumanth C.H.M. (2020). TGA and DSC analysis of vinyl ester reinforced by Vetiveria zizanioides, jute and glass fiber. Mater. Today Proc..

[70] Junior V.D.L., Leão R.M., Steier V.F., Da Luz S.M. (2020). Influence of cure agent, treatment and fibre content on the thermal behaviour of a curaua/epoxy prepreg. Plast. Rubber Compos..

[71] Sen A.K., Kumar S. (2010). Coir-fiber-based fire retardant nano filler for epoxy composites. J. Therm. Anal. Calorim..

[72] Siregar J.P., Salit M.S., Rahman M.Z.A., Dahlan K.Z.H.M. (2011). Thermogravimetric analysis (TGA) and differential scanning calometric (DSC) analysis of pineapple leaf fibre (PALF) reinforced high impact polystyrene (HIPS) composites. J. Sci. Technol..

[73] Ramachandran M., Bansal S., Raichurkar P. (2016). Scrutiny of jute fiber poly-lactic acid (PLA) resin reinforced polymeric compo-site. J. Text. Assoc..

[74] Sider I., Nassar M. (2021). Chemical treatment of bio-derived industrial waste filled recycled low-density polyethylene: A comparative evaluation. Polymers.

[75] Chandekar H., Chaudhari V., Waigaonkar S., Mascarenhas A. (2019). Effect of chemical treatment on mechanical properties and water diffusion characteristics of jute-polypropylene composites. Polym. Compos..

[76] Filho F.C.G., Luz F.S., Nascimento L.F.C., Satyanarayana K.G., Drelich J.W., Monteiro S.N. (2020). Mechanical properties of *Boehmeria nivea* natural fabric reinforced epoxy matrix composite prepared by vacuum-assisted resin infusion molding. Polymers.

[77] Pupure L., Varna J., Joffe R., Berthold F., Miettinen A. (2018). Mechanical properties of natural fiber composites produced using dynamic sheet former. Wood Mater. Sci. Eng..

[78] Revol B.P., Vauthier M., Thomassey M., Bouquey M., Ruch F., Nardin M. (2021). Design of experience to evaluate the Interfacial compatibility on high tenacity viscose fibers reinforced Polyamide-6 composites. Compos. Sci. Technol..

