# Supplementary material for: Physical and Mechanical Characterization of Titica Vine (Heteropsis flexuosa) Incorporated Epoxy Matrix Composites
Source: Polymers (Basel). 2021 Nov 24;13(23):4079. doi: 10.3390/polym13234079 (PMC8659021; doi:10.3390/polym13234079)
Supplement: Supplementary file 1 [file polymers-13-04079-s001.zip › polymers-1434382-supplementary.pdf]

polymers

MDPI

Supplementary Materials

Physical and Mechanical Characterization of Titica Vine (*Heteropsis flexuosa*) Incorporated Epoxy Matrix Composites

Juliana dos Santos Carneiro da Cunha [1], Lucio Fabio Cassiano Nascimento [1], Fernanda Santos da Luz [1], Sergio Neves Monteiro [1,*], Maurício Ferrapontoff Lemos [2], Cristina Gomes da Silva [3], and Noan Tonini Simonassi [4]

[1] Department of Materials Science, Military Institute of Engineering – IME, Praça General Tibúrcio, 80, Urca, Rio de Janeiro, 22290-270, Brazil; julianasccunha@gmail.com (J.d.S.C.d.C.); lucio@ime.eb.br (L.F.C.N.); fsl.santos@gmail.com (F.S.d.L.); snevesmonteiro@gmail.com (S.N.M.)
[2] Brazilian Navy Research Institute – IpqM, Materials Technology; Rua Ipiru, 02, Cacuia, Rio de Janeiro, 21931-095, Brazil; mauricio.lemos@marinha.mil.br (M.F.L.)
[3] Department of Materials Science, Federal University of Amazonas – UFAM, Avenida General Rodrigo Octávio Jordão Ramos, 1200 – Coroado 1, Manaus, 69067-005, Brazil; cristinagomes.ufam@gmail.com (C.G.d.S.)
[4] State University of the Northern Rio de Janeiro – UENF, Avenida Alberto Lamego, 2000, Campos dos Goytacazes, 28013-602, Brazil; noantoninisimonassi@gmail.com (N.T.S.)

* Correspondence: snevesmonteiro@ime.eb.br

1. Water Absorption

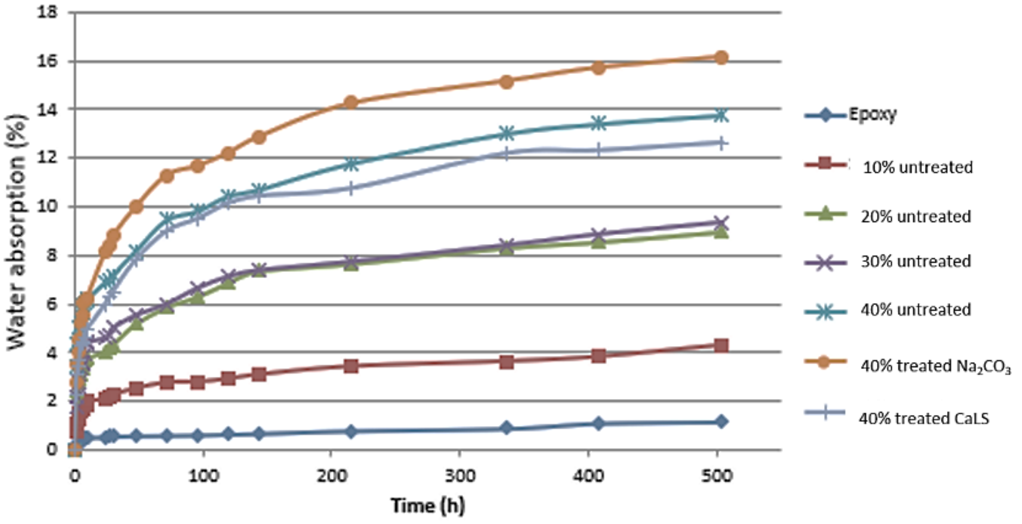

Figure S1. water absorption curve for neat epoxy as well as untreated and treated TVF composites.

2. Charpy and Izod Impact Test

Figure S2 presents the Charpy and Izod average impact energy results for neat epoxy and untreated composites with different volumetric fractions of TVF. For the Charpy test, Figure S3 (a), it was possible to observe that the addition of 40 vol% TVF increased 18% in

the absorbed energy in relation to the control group of neat epoxy, while the Izod test in Figure S2 (b) increased 28%.

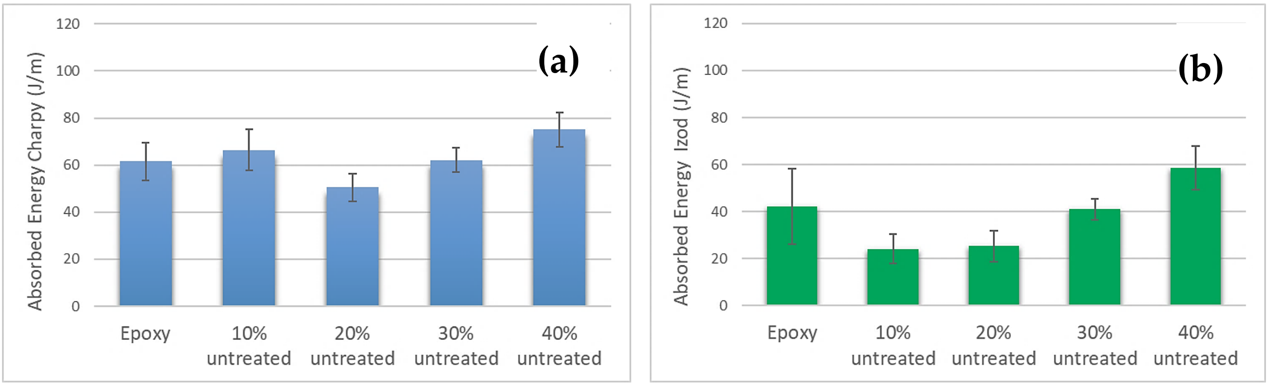

Figure S2. Variation of the absorbed impact energy of the composites in relation to the volume fraction of untreated TVF for (a) Charpy; and (b) Izod tests.

The composite with TVF treated with Na_2CO_3 and CaLS impact test results are shown in Figure S3. The same trend was observed for untreated fiber composites in Figure S2. The increase in fiber content influenced the higher average absorbed energy for the samples with 40 vol% TVF in both tests.

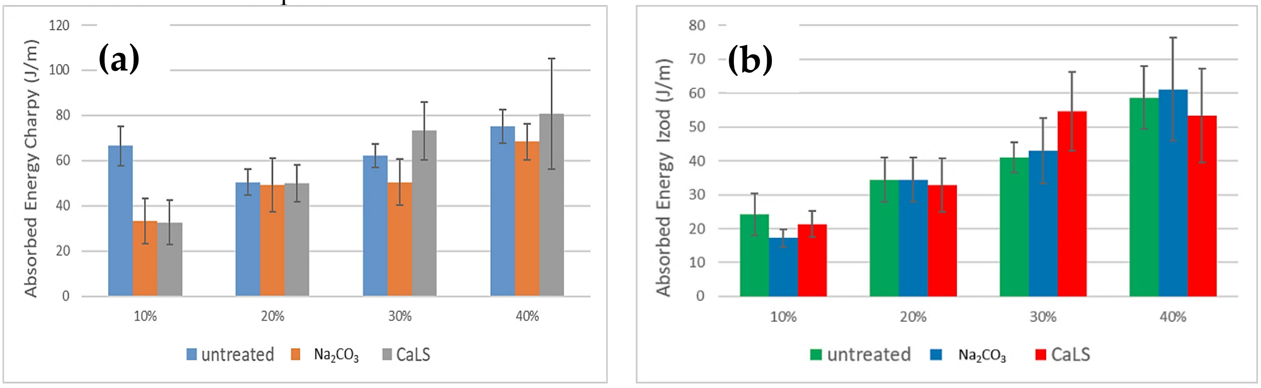

Figure S3. Variation of the absorbed impact energy of the composites in relation to the volumetric fraction of untreated and treated TVF for the (a) Charpy; (b) Izod.

To confirm the effect provided by TVF, the Charpy and Izod impact energy absorption results of the composites were statistically analyzed using the Weibull method. It was chosen for analysis all groups with untreated fibers and particularly groups of 40 vol% TVF both untreated fibers and treated with Na_2CO_3 and CaLS. The choice of this specific group (40 vol%) was due to the fact that it presented the highest impact resistance in both tests.

The standard deviation values of the compositions may compromise the degree of certainty about whether the fibers acted as reinforcement in the matrix, as well as whether the treatments were effective or not. Therefore, analysis of variance (ANOVA) and Tukey test were performed to verify the occurrence of significant difference between the Charpy and Izod absorbed energy results for the same groups proposed by the Weibull analysis.

Tables S1 and S2 present the ANOVA of the results of Charpy and Izod impact resistance for untreated 40 vol% TVF composites, respectively.

Table S1. ANOVA for the absorbed impact energy Charpy and Izod of composites with different volume fractions of untreated TVFs.

	0 - 40% untreated Charpy					0 - 40% untreated Izod				
**Variation Causes**	**DF**	**SS**	**MS**	**F_{calc}**	**$F_{critical}$**	**DF**	**SS**	**MS**	**F_{calc}**	**$F_{critical}$**
**Treatment**	4	2540.55	635.14	12.60	2.64	4	9617.28	2404.32	27.43	2.54
**Residue**	35	1764.94	50.43			55	4821.56	87.66		
**Total**	39	4305.50				59	14438.84			

Table S2. ANOVA for the absorbed impact energy Charpy and Izod of composites with 40 vol% of untreated and treated TVFs.

	40 vol% untreated / treated Charpy					40 vol% untreated / treated Izod				
**Variation Causes**	**DF**	**SS**	**MS**	**F_{calc}**	**$F_{critical}$**	**DF**	**SS**	**MS**	**F_{calc}**	**$F_{critical}$**
**Treatment**	2	607.49	303.74	1.26	3.47	2	380.77	190.39	1.12	3.28
**Residue**	21	5043.6	240.17			33	5606.65	169.90		
**Total**	23	5651.05				35	5987.42			

Table S3 and S4 present the values of the honestly significant difference (HSD) obtained by the Tukey test for the Charpy and Izod results, respectively.

Table S3. HSD as measured by Tukey test for the Charpy absorbed impact energy of composites with volume different fractions of untreated TVFs.

Sample	Epoxy	10% untreated	20% untreated	30% untreated	40% untreated
Epoxy	0.00	4.92	**11.07**	0.62	**13.53**
10% untreated	4.92	0.00	**15.99**	4.31	8.61
20% untreated	**11.07**	**15.99**	0.00	**11.69**	**24.61**
30% untreated	0.62	4.31	**11.69**	0.00	**12.92**
40% untreated	**13.53**	8.61	**24.61**	**12.92**	0.00

Table S4. HSD as measured by Tukey test for the Izod absorbed impact energy of composites with different fractions of volume of untreated TVFs.

Sample	Epoxy	10% untreated	20% untreated	30% untreated	40% untreated
Epoxy	0.00	**18.04**	**16.81**	1.23	**16.4**
10% untreated	**18.04**	0.00	1.23	**16.81**	**34.45**
20% untreated	**16.81**	1.23	0.00	**15.58**	**33.22**
30% untreated	1.23	**16.81**	**15.58**	0.00	**17.63**
40% untreated	**16.4**	**34.45**	**33.22**	**17.63**	0.00

Publisher's Note: MDPI stays neutral with regard to jurisdictional claims in published maps and institutional affiliations.

© 2020 by the authors. Licensee MDPI, Basel, Switzerland. This article is an open access article distributed under the terms and conditions of the Creative Commons Attribution (CC BY) license (http://creativecommons.org/licenses/by/4.0/).
